# Small cell lung cancer (SCLC): At the door of targeted therapies

**DOI:** 10.17305/bb.2025.13195

**Published:** 2025-08-29

**Authors:** Krešimir Tomić, Semir Vranić

**Affiliations:** 1 Department of Oncology, University Hospital Center Mostar, Mostar, Bosnia and Herzegovina; 2 College of Medicine, QU Health, Qatar University, Doha, Qatar

Small cell lung cancer (SCLC) is a neuroendocrine lung neoplasm predominantly associated with tobacco exposure [1]. It constitutes approximately 15% of all lung cancer cases, with around 150,000 new diagnoses globally each year. For decades, SCLC has represented stagnation in thoracic oncology. Unlike non-SCLC (NSCLC), which has experienced significant advancements through targeted and immunotherapy treatments that lead to improved survival rates, SCLC has remained a therapeutic challenge. This stagnation can be attributed to several factors: rapid tumor proliferation, aggressive biological behavior, and late-stage diagnosis in most patients. Current screening methods do not facilitate early detection, and even in limited-stage SCLC (LS-SCLC), surgical intervention is viable in fewer than 5% of cases [2]. Consequently, the majority of patients with LS-SCLC receive concurrent chemoradiotherapy, which remains the cornerstone of curative-intent treatment.

For decades, platinum-etoposide has been regarded as the gold standard for first-line therapy in extensive-stage SCLC (ES-SCLC), resulting in an overall survival (OS) of less than 12 months [2]. This stagnation in treatment options has led to SCLC being classified as an orphan disease and a challenging landscape for drug development. Prior to the advent of immunotherapy, the only notable advances in ES-SCLC treatment were achieved through radiotherapy and prophylactic cranial irradiation (PCI) or MRI brain surveillance. Consolidative thoracic radiotherapy has been shown to improve survival in patients who respond to chemotherapy [3]. Given that more than 50% of patients ultimately develop intracranial metastases, PCI has been employed to mitigate the risk of symptomatic brain involvement [4]. Additionally, active MRI surveillance presents an alternative strategy for patients who do not receive PCI [5].

**Figure 1.**
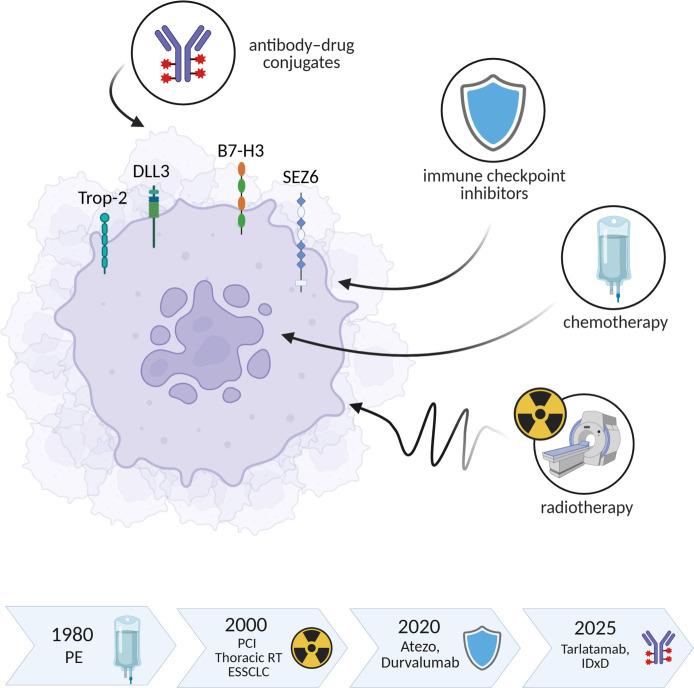
**Therapeutic evolution in SCLC from 1980 until mid-2025.** A schematic SCLC cell highlights major druggable surface antigens—DLL3 and B7-H3 (clinically validated targets) and Trop-2 and SEZ6 (emerging ADC targets)—and treatment platforms: ADC, ICI, CHT, and RT. The lower timeline summarizes milestones: ∼1980 introduction of platinum–etoposide; ∼2000 adoption of PCI and consolidative thoracic RT in ES-SCLC; 2020 addition of atezolizumab or durvalumab to first-line chemotherapy; 2025 emergence of the DLL3-directed T-cell engager tarlatamab and the B7-H3–directed ADC I-DXd for relapsed ES-SCLC. The figure emphasizes the shift from uniform chemotherapy toward biomarker-guided precision therapy. Abbreviations: ADC: Antibody–drug conjugates; Atezo: Atezolizumab; ICI: Immune checkpoint inhibitors; CHT: Chemotherapy; RT: Radiotherapy; PE: Cisplatin/etoposide; PCI: Prophylactic cranial irradiation; ES-SCLC: Extensive stage small cell lung cancer; IDxD: Ifinatamab deruxtecan; SCLC: Small cell lung cancer.

The landscape of SCLC has undergone significant transformation due to two key developments. First, the identification of four molecular subtypes of SCLC, characterized by distinct transcriptional signatures, has laid the groundwork for a more personalized treatment approach: SCLC-A (ASCL1/ASH1), SCLC-N (NEUROD1), SCLC-P (POU2F3), and the inflammatory subtype SCLC-I, which appears most likely to benefit from immunotherapy using immune checkpoint inhibitors (ICIs) [6, 7]. Second, the advent of ICIs has represented the most meaningful advancement in SCLC treatment in over three decades (Figure 1). Although the addition of atezolizumab or durvalumab to chemotherapy in ES-SCLC resulted in only a modest median survival increase of approximately two months, it revealed an unprecedented phenomenon: a survival tail of long-term responders [8, 9]. Building on this initial finding, subsequent studies have indicated additional benefits for both ES-SCLC and, for the first time, LS-SCLC. The IMforte study demonstrated that incorporation of lurbinectedin with atezolizumab as maintenance therapy following chemoimmunotherapy in ES-SCLC enhances OS [10]. In contrast, the ADRIATIC study represents a significant breakthrough in LS-SCLC, showing that durvalumab consolidation after concurrent chemoradiotherapy improved OS by an impressive 22 months [11].

Despite the notable chemosensitivity of SCLC, treatment responses are often transient, leading to nearly universal relapse. Patients experiencing relapse after six months may consider platinum rechallenges; however, those relapsing within 90 days face a significantly poor prognosis. In the second-line treatment setting, topotecan and the CAV regimen (cyclophosphamide, doxorubicin, and vincristine) have historically been the primary standard therapies. Meanwhile, newer agents such as lurbinectedin and amrubicin have not demonstrated an OS benefit compared with the previously established topotecan or CAV regimen.

Replacing a one-size-fits-all approach with a precise, targeted strategy necessitates the identification of a biomarker that is overexpressed in SCLC while being minimally present in normal cells. A prime example of such a biomarker is Delta-like ligand 3 (DLL3). DLL3 is an inhibitory Notch ligand that promotes cancer growth, invasion, and metastasis by modulating Notch signaling pathways, rendering it a compelling therapeutic target [12]. DLL3 is significantly overexpressed in various neuroendocrine tumors, particularly SCLC [13]. High DLL3 expression on the surface of cancer cells has been reported in over 80% of SCLC cases, suggesting that target-directed therapies, such as antibody-drug conjugates (ADCs) and T-cell engagers, may be viable options [1, 14, 15].

Tarlatamab, a bispecific T-cell engager (BiTE®), recruits cytotoxic T cells to selectively target DLL3-expressing tumor cells, leading to the destruction of cancer cells. In the randomized phase 3 DeLLphi-304 study, tarlatamab extended the median OS to 13.6 months, compared to 8.3 months for standard-of-care chemotherapy in second-line extensive-stage SCLC (HR 0.60, *P* < 0.001) [16]. Notably, DLL3 expression was not a prerequisite for trial participation, and the study population included patients with platinum-resistant disease, brain metastases, and a majority (71%) having prior exposure to immunotherapy. The survival benefit was consistent across subgroups, including these high-risk populations. Toxicity was manageable, with grade ≥3 adverse events occurring less frequently than with chemotherapy. Additionally, new immune-mediated toxicities were observed, including cytokine release syndrome in 56% of patients (predominantly grade 1–2) and immune effector cell-associated neurotoxicity syndrome (ICANS) in approximately 6%. Based on these transformative results, tarlatamab is now regarded as a new standard of care for the second-line treatment of ES-SCLC.

B7 homolog 3 (B7-H3) also known as CD276, a member of the B7 family of immune checkpoint regulators, represents a promising target for the treatment of SCLC. This protein is integral to tumor growth, metastasis, immune evasion, and resistance to therapies [17]. B7-H3 expression has been documented in various cancers, including SCLC [18, 19], leading to its identification as a potential therapeutic target. High levels of B7-H3 expression correlate with poor prognosis; in contrast to DLL3, which exhibits variable expression across molecular subtypes, B7-H3 is consistently overexpressed across all four SCLC subtypes [20]. This biological characteristic supports the rationale for a favorable response to B7-H3-directed therapies.

The phase 2 IDeate-Lung01 study provided significant evidence for this rationale, demonstrating exceptional efficacy of the ADC ifinatamab deruxtecan (I-DXd) in heavily pretreated ES-SCLC [21]. Among patients who had undergone at least two prior lines of therapy, 76% of whom had prior immunotherapy, the 12 mg/kg dose cohort achieved a confirmed objective response rate (ORR) of 54.8%, a median progression-free survival (PFS) of 5.5 months, and a median OS of 11.8 months. Notably, I-DXd also produced clinically meaningful responses in patients with brain metastases, a subgroup historically associated with poor outcomes. Based on these landmark findings, the U.S. Food and Drug Administration (FDA) granted I-DXd breakthrough therapy designation for heavily pretreated patients with ES-SCLC [22].

Responses to both tarlatamab and I-DXd were observed regardless of DLL3 and B7-H3 expression levels [16, 23]. In contrast, when evaluating predictors of chemoimmunotherapy efficacy, DLL3 expression did not correlate with treatment response, while B7-H3 did [24]. Elevated B7-H3 expression is associated with reduced survival and diminished CD8^+^ T-cell function, highlighting its dual role as both a therapeutic target and a potential prognostic biomarker.

Recent advances are significantly transforming the therapeutic landscape of SCLC. Therapies targeting DLL3 and B7-H3 represent complementary breakthroughs, demonstrating unprecedented efficacy in second- and later-line treatments for ES-SCLC. Additionally, ADCs targeting the transmembrane glycoprotein Trop2 and seizure protein 6 (SEZ6) are currently under development, indicating that the previously sparse pipeline for SCLC is beginning to fill with viable treatment options (Figure 1).

Nonetheless, several key challenges persist, including the optimization of treatment sequencing and combinations to counteract rapid resistance, as well as the expansion of these strategies into first-line settings. Equally important are approaches to manage the unique toxicities of BiTE molecules and the development of predictive biomarkers for improved patient selection.

Importantly, the trajectory of SCLC treatment is shifting. After decades of limited progress, the field is experiencing a notable transformation: relapsed SCLC is moving from a reliance on uniform chemotherapy toward a precision medicine paradigm (Figure 1). For the first time, the narrative surrounding SCLC is characterized not by despair but by cautious optimism.
